# Spontaneous hemorrhagic strokes during pregnancy: case report and review of the literature

**DOI:** 10.11604/pamj.2014.19.372.5422

**Published:** 2014-12-11

**Authors:** Meriem Laadioui, Wail Bouzoubaa, Sofia Jayi, Fatima Zohra Fdili, Hakima Bouguern, Hikmat Chaara, My Abdelilah Melhouf

**Affiliations:** 1 Sidi Mohammed Ben Abdellah University, Department of Gynecology and Obstetrics, University Hospital of Fez, Morocco

**Keywords:** Hemorrhagic stroke, Pregnancy, Post partum, Brain scan

## Abstract

Hemorrhagic stroke is responsible for significant morbidity and mortality. Postpartum and pregnancy are risk period. Only urgent care in intensive care units may improve prognosis. We report the case of 22 years old's Morrocan, who presented to our department with an intense headache headset followed a few hours later by consciousness disorder. Clinical examination at admission has objectified a woman obsessed with a GCS 13, normotensive, the labstix is negative. A brain scan was performed showing left temporal intra parenchymal hematoma with ventricular flooding and subfalcine herniation. An external ventricular shunt was made. The patient was extubated on day 2 of hospitalization, with progressive neurological improvement. Concerning obstetrical care, the pregnancy has evolved harmoniously without any growth retardation or other abnormalities, with full-term vaginal delivery of a healthy 3kg200 baby. although Hemorrhagic stroke during pregnancy is rare, the prognosis is reserved. An adequate care in intensive care unit is required.

## Introduction

Although rare during pregnancy, the hemorrhagic strokes are responsible for significant maternal mortality. These accidents usually occur in the last days of pregnancy and the first few weeks postpartum. The etiological investigation must be as rigorous as outside the pregnancy period, only a rational diagnostic approach can provide an appropriate therapeutic management. Pregnancy does not contraindicate complementary examinations, including those using X-rays if they are deemed necessary. Further studies are needed to specify the cerebrovascular risk for a woman who had a stroke associated with pregnancy or postpartum.

## Patient and observation

**Figure 1:**
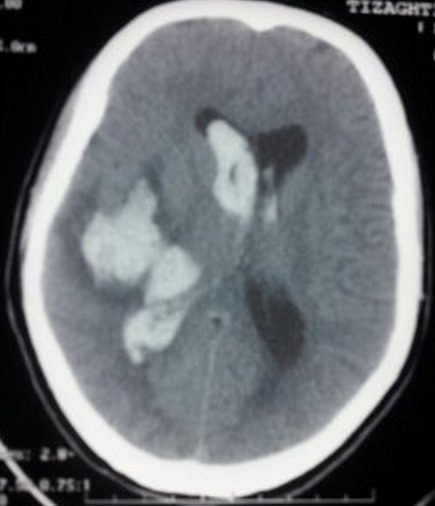
Temporal intra parenchymal hematoma with ventricular flooding and subfalcine herniation

Mrs. N. T parturient aged 22, without any significant medical history, namely no hypertension or smoking, second pregnancy, having a living 8 months child, the current pregnancy is estimated at 7 months, unmonitored, normal, the day of her admission, the patient had sudden intense headache headset followed a few hours later by consciousness disorder. Clinical examination at admission has objectified a woman obsessed with a GCS 13, normotensive, the labstix is negative, obstetrical examination revealed a uterine height corresponding to gestational age, and BCFs are present and regular without uterine contractions or bleeding. Given this symptomatology, laboratory tests including preeclampsia were requested, whose results were normal, a brain scan was performed showing left temporal intra parenchymal hematoma with ventricular flooding and subfalcine herniation (Figure 1). Given this table, the surgical indication of an external ventricular shunt was present. The patient was transferred to intensive care; she was put under antiepileptic treatment. During her stay, she received an angio-MRI which has not objectified arterio-venous malformation. The follow up scanner done 48h after admission revealed that the hematoma was in resorption with regression of the ventricular flooding. The patient was extubated on day 2 of hospitalization, with progressive neurological improvement. Concerning obstetrical care, the pregnancy has evolved harmoniously without any growth retardation or other abnormalities, with full-term vaginal delivery of a healthy 3 kg 200 baby.

## Discussion

Stroke during pregnancy is a rare event (23/474 women of childbearing age). Strokes represent 4 to 11% of the causes of maternal deaths. Sudden onset of this disease is a therapeutic emergency, only early care within a specialized care unit helps to reduce the morbidity and mortality. It is secondary to intra-parenchymal rupture of an intracranial vessel causing hematoma to be responsible for brain lesions that are explained by arterial ischemia or compressive failure of blood supply artery broken with release of molecules nerve [1]. It is usually an intracerebral hematoma, subarachnoid hemorrhage or cerebral subarachnoid hemorrhage [2]. Currently, it is difficult to relate the effects of pregnancy and postpartum to a stroke occurrence, however it seems that the post partum and pregnancy are risk periods. According to Kittner and all, the ischemic and hemorrhagic stroke is more likely to occur during the puerperal period and not the pregnancy [3, 4]. Hypertension is a major risk factor for stroke during pregnancy and the postpartum period, in fact, 44% of HIP pregnancy and postpartum are associated with preeclampsia and eclampsia syndrome. [5] The vascular risk factors of stroke among young people, especially smoking and obesity are often found [6]. However, none of the above mentioned factors have been found in the case of our patient, which allows us to think that the physiological changes associated with pregnancy may increase the risk of hemorrhagic stroke, namely the one secondary to rupture of an arterial aneurysm or arterio-venous malformation. The diagnosis of stroke is based on a brain scan without injection of contrast medium, which allows the diagnosis of a cerebral hematoma or cerebral hemorrhage in the acute phase [7]. This examination is not counter-indicated in gravid-postpartum state because the radiation doses of a brain scan are well below the threshold of risk of fetal exposure [8]. Although MRI is the chosen modality of medical imaging explorations in the case of pregnant women, its safety in the first quarter is not currently demonstrated. This examination remains of interest to research as a possible cause (dissection, atheroma) [8]. The therapeutic care of hemorrhagic stroke occurring during pregnancy or postpartum should be identical to that conducted outside these periods, particularly regarding symptomatic and preventive measures. Emphasis should be on the control of blood pressure and the research and correction of causative factors. It is mainly to find out intracerebral vascular malformation, which may require urgent surgical care [5]. The maintenance of pregnancy is often compromised in the most serious cases, requiring admission to ICU or neurosurgical intervention to evacuate the hematoma or exclude a vascular malformation cases [2].

Whether it is the case of a hemorrhagic brain strokes or medullar ones, where there is a serious array of intracranial pressure or compression of the spinal cord, cesarean section is mandatory in late pregnancy before any necessary urgent neurosurgical treatment [9]. When a vascular malformation or aneurysm arterio-venous malformation type is diagnosed, the decision must be multidisciplinary involving neurologists, anesthesiologists, neurosurgeons and neuro-radiologists. The choice will be between endovascular treatment and first neurosurgical lesion [2]. In the case of our patient, the stroke occurred in the 7th month of pregnancy; our attitude was conservative after consultation with the resuscitators. In the absence of obvious etiology of stroke and the favorable evolution, vaginal delivery is accepted. Fetal morbidity seems to be more associated with the occurrence of gestational intra-parenchymal hemorrhage, particularly due to the induced prematurity it generates. However, there is no data in the literature that allow us to confirm this observation [5]. A study conducted by Aya and all has revealed 4 neonatal deaths in 23 pregnancies including 2 related to maternal mortality, 2 related to abortion. The prognosis of hemorrhagic strokes occurring during pregnancy or postpartum seems to be more severe than ischemic strokes. Only an adequate care in intensive care environment can ensure a better prognosis.

## Conclusion

Hemorrhagic stroke is a therapeutic emergency. During pregnancy the maternal and fetal prognosis are involved. The multidisciplinary management allows you to find the best compromise between the requirements imposed by the state of the mother and those related to the presence of fetus.
